# Evaluation of a New Lipase from *Staphylococcus* sp. for Detergent Additive Capability

**DOI:** 10.1155/2013/374967

**Published:** 2013-09-10

**Authors:** Mamta Chauhan, Rajinder Singh Chauhan, Vijay Kumar Garlapati

**Affiliations:** Bioprocess Engineering Laboratory, Department of Biotechnology and Bioinformatics, Jaypee University of Information Technology, Waknaghat, Himachal Pradesh 173 234, India

## Abstract

Lipases are the enzymes of choice for laundry detergent industries owing to their triglyceride removing ability from the soiled fabric which eventually reduces the usage of phosphate-based chemical cleansers in the detergent formulation. In the present study, a partially purified bacterial lipase from *Staphylococcus arlettae* JPBW-1 isolated from the rock salt mine has been assessed for its triglyceride removing ability by developing a presoak solution so as to use lipase as an additive in laundry detergent formulations. The effects of selected surfactants, commercial detergents, and oxidizing agents on lipase stability were studied in a preliminary evaluation for its further usage in the industrial environment. Partially purified lipase has shown good stability in presence of surfactants, commercial detergents, and oxidizing agents. Washing efficiency has been found to be enhanced while using lipase with 0.5% nonionic detergent than the anioinic detergent. The wash performance using 0.5% wheel with 40 U lipase at 40°C in 45 min results in maximum oil removal (62%) from the soiled cotton fabric. Hence, the present study opens the new era in enzyme-based detergent sector for formulation of chemical-free detergent using alkaline bacterial lipase.

## 1. Introduction

Lipases (triacylglycerol acyl hydrolase, E.C. 3.1.1.3.) are ubiquitous enzymes with industrial potential of synthesizing structural triglycerides, which serves as detergents and emulsifiers in nutrition and cosmetics [1]. Detergent enzymes constitute about 32% of the total world-wide industrial enzyme production [2]. Thermal stability is a major requirement for a commercial enzyme such as lipase which would allow enzymatic reaction to being performed at a higher temperatures and would be helpful to increase conversion rates and substrate solubility. The importance of alkaline and thermostable lipases for different applications has been growing rapidly [3]. The increasing demand of alkaline lipases as a detergent additive is mainly due to its affiliation with the nonphosphate detergents. Ideally, alkaline lipases in a detergent formulation should be stable over a broad range of temperature, pH and compatible with surfactants and oxidizing agents at lower concentrations with broad substrate specificity [4]. 

The detergent industries are relying on recombinant lipases (Lipex and Lipolase from Novozymes) for formulation of biodetergents due to their stability in presence of harsher detergent formulation ingredients such as surfactants and oxidizing agents [5]. Researchers are in continuous search of lipases from indigenous extremophilic regions for better application in laundry detergent industry. Among different sources (fungal, yeast, and bacterial), bacterial lipases received much attention for their ability to function in extreme environments of temperature, pH, surfactant and oxidizing agents [6]. Rathi et al. [7] showed the application of bacterial lipase *Burkholderia cepacia* as an additive in detergent formulation which exhibits better stability towards commercial detergents and oxidizing agents in comparison to commercial Lipolase. In another study, Thirunavukarasu et al. [8] have shown the use of *Cryptococcus* sp. S-2 lipase in detergent formulation and optimized washing conditions through response surface methodology. Moreover, bacterial lipases added to household detergents reduce or replace synthetic detergents, which have been considering as an environmental pollutants [9]. Ideally, alkaline lipases are suitable candidates as a detergent additive for formulating a presoak formulation in detergent industry [10]. 

Hence, in the present study we have made an attempt to assess the triglyceride removing ability of an alkaline bacterial lipase produced by *Staphylococcus arlettae *JPBW-1. Preliminarily,lipase compatibility studies with surfactants, oxidizing agents, and readily available commercial detergents have been checked and later tested for its washing efficiency for olive oil removal from soiled cotton fabric. 

## 2. Materials and Methods

### 2.1. Microorganisms, Chemicals, and Reagents

*Staphylococcus arlettae* JPBW-1 was used for the lipase production, which was isolated from the one and only hotspring of India, Darang, HP, and deposited in MTCC, Chandigarh (India), as *Staphylococcus arlettae* JPBW-1 MTCC5589 maintained on Luria agar slants at 4°C.

*Olive Oil*. Olive oil used was the brand of Sos Cuetara, S.A. Figaro.

*Surfactants*. Surfactants used were commercial grade products. Tween 80 as a nonionic surfactant and commercial detergents of Indian market, namely, Ariel, Tide, Active wheel and Nirma (Procter and Gamble Home Products Ltd.), Rin Magic and Surf Excel (Hindustan Lever Ltd.) and sodium dodecyl sulphate (SDS) as anionic surfactants. *p-*Nitrophenyl palmitate (*p-*NPP) was procured from Sigma, USA. 

### 2.2. Lipase Production and Partial Purification

Bacterial lipase was produced through submerged fermentation and partially purified through ammonium sulphate precipitation (60%) as reported by Mamta et al., 2013 [11]. The lipase has a pH optima of 11.0 and active under broad temperature range of 25–100°C [11]. 

### 2.3. Lipase Assay

Lipase activity was determined using *p*-NPP as a substrate [12]. One unit (U) of lipase activity was expressed as the amount of enzyme that liberates one micromole of *p*-nitrophenol per minute under the assay conditions. 

### 2.4. Compatibility of Lipase with Surfactants and Commercial Detergents

To investigate the lipase compatibility with various surfactants and commercial detergents, respective surfactants and detergents were added to the reaction mixture at a concentration of 7 mg/mL and assayed under standard conditions and expressed in terms of percent relative activity. The endogenous lipases present in these detergents were inactivated by heating the diluted detergents for 1 h at 65°C prior to the addition of enzyme preparation. To determine the stability, an aliquot of enzyme sample (50 U/mL) was incubated with equal volume of detergent solution (7 mg/mL of respective detergent) in Tris-HCl buffer (0.1 M, pH 8.0) for 1 h at 30°C. The relative activity (%) of each sample was determined and compared with the control without detergent. The relative activity of control was defined as the enzyme activity without detergent, incubated under the similar conditions, and was taken as 100%.

### 2.5. Compatibility of Lipase with Oxidizing Agents

Lipase (50 U/mL) compatibility in the presence of oxidizing agents was determined in Tris-HCl buffer (0.1 M, pH 8.0) containing 0.5–2.0% (v/v or w/v) of hydrogen peroxide, sodium perborate, and sodium hypochlorite for 1 h at 30°C, and relative activity was estimated and compared with the control without oxidizing agent. The relative activity of control was defined as the enzyme activity without oxidizing agent, incubated under the similar conditions, and was taken as 100%.

### 2.6. Preparation of Olive Oil Soiled Cotton Fabric

Defatting of cotton fabric (5 cm × 10 cm) supposed to be soiled was carried out by boiling in choloroform for 4 h and was repeated thrice. Soiling of defatted cotton fabric was done twice with micropipette by spotting olive oil in benzene solution (0.5 mL, 100 mg/mL Conc). 

### 2.7. Washing Solution Preparation

Washing solutions of four kinds and its composition for preparing 100 mL of respective washing solution were shown in Table 1. Solution B-D-L, consists of buffer, surfactant and lipase solutions, was prepared by taking the measured buffer and surfactant solutions in Erlenmeyer flask with ground stopper. Preheat (37°C for 10 min) the contents of the flask before adding the lipase solution. B-L, B-D and B solutions were also prepared in the same manner. The final volume of the respective washing solutions was adjusted to 100 mL with distilled water. For selecting the best process conditions, initially washing compositions of Table 1 were used. After selection of one best process condition through changing one variable at a time approach, the best condition was used for selection of the next process condition by utilizing the proportion of Table 1 for making the detergent solutions. 

### 2.8. Washing Procedure and Olive Oil Determination

Washing of soiled fabric dipped in respective washing solutions was done by shaking the flask contents at 100 rpm at 37°C for 20 min. After 20 min, the washed soiled fabric was rinsed thrice with 100 mL distilled water for 2 min at 37°C and air dried. For selection of better washing conditions, the washing process was done at different temperatures and time intervals using different Concs. of detergent and lipase activities. The olive oil was extracted (from the respective washed cotton fabric) with petroleum ether for 6 h in Soxhlet extractor. After complete evaporation of petroleum ether from the extract, the weight of oilve oil was determined.

 The % of oil removal was calculated by using the following formula

(1) $$\begin{array}{c} \text{Removal}\;\left(\%\right)=\frac{\left(W_{i}-W_{r}\right)}{W_{r}}\times 100, \end{array}$$

where *W*_*i*_ is weight of total olive oil before washing (mg) and *W*_*r*_ is weight of total olive oil after washing (mg). 

## 3. Results and Discussion

### 3.1. Compatibility of Lipase with Surfactants and Commercial Detergents

**Figure 1:**
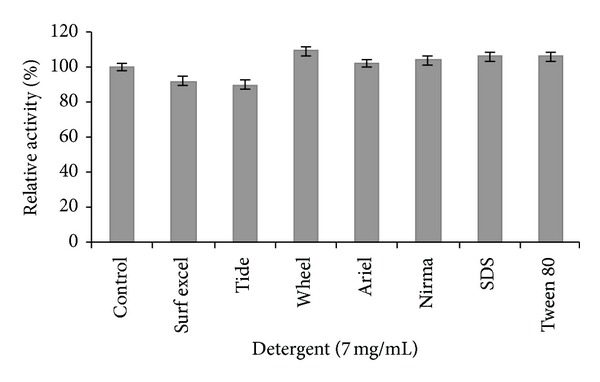
Compatibility of *S. arlettae* lipase with surfactants and detergents. For the control, lipase was incubated with buffer devoid of surfactants and detergents and its activity was taken as 100%. All values are represented as mean ± sd of three replications.

For effective use under harsh detergent industry conditions, lipolytic enzyme must be compatible and stable with all commonly used detergent formulation ingredients such as surfactants [13]. The *S. arlettae *lipase has been shown excellent compatibility and stability in presence of anionic and nonionic surfactants as well as in commercial detergents. The enzyme showed increased stability in presence of SDS and Tween 80, and similar results were also reported for lipases from *Aspergillus* sp. and *Rhizopus* sp. [14, 15]. Among various detergents tested, better enhanced lipase activity has been observed with wheel and an 6% enhancement with SDS over control. (Figure 1). However, the lipase activities of *Ralstonia pickettii* [16] and *Aspergillus carneus* were inhibited in the presence of SDS [17] while the activity was increased in case of *H*. *lanuginosa* lipase [18].

### 3.2. Lipase Compatibility with Oxidizing Agents

**Figure 2:**
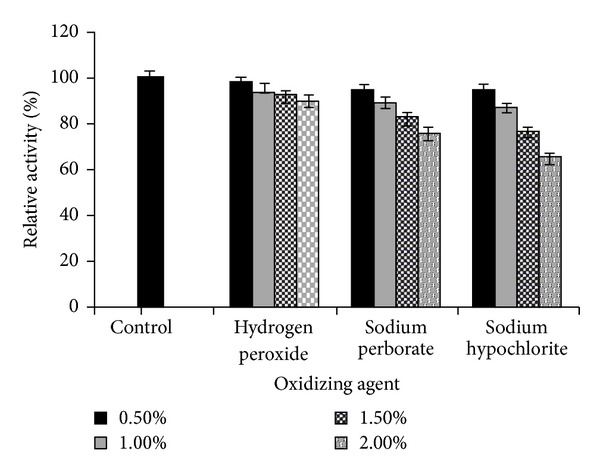
Compatibility of *S. arlettae* lipase with oxidizing agents. For the control, lipase was incubated with buffer alone without oxidizing agent and its activity was taken as 100%. All values are represented as mean ± sd of three replications.

Bleach stability of lipase was also checked in presence of hydrogen peroxide, sodium hypochlorite, sodium perborate, and sodium peroxide. The lipase was highly stable towards oxidizing agents at 1.5% concentration for 1 h at 30°C and it retained 92% of activity even at 2.0% concentration of hydrogen peroxide, while activity was gradually decreased with an increase in Conc. of sodium perborate and sodium hypochlorite from 1.0 to 2.0% (Figure 2). Remarkably, the present lipase exhibited better resistance towards strong oxidizing agents especially hypochlorite (95% activity at 1.0% concentration) compared to the relative activity of Lipolase (Novozymes, Denmark), which exhibited 43% activity after 1 h treatment as reported by Rathi et al. [7]. Therefore, the higher stability in presence of oxidizing agents makes this alkaline lipase is an ideal enzyme that can be incorporated into any detergent formulation for enhanced results.

### 3.3. Effect of Detergent and Its Concentration on Oil Removal

**Figure 3:**
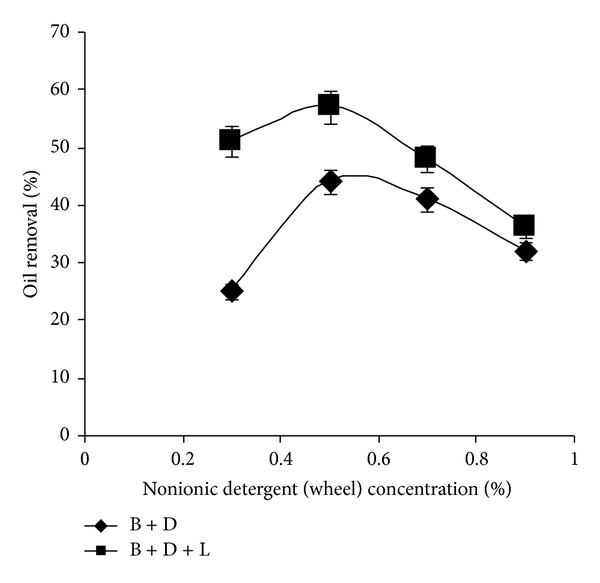
Effect of detergent and its concentration on oil removal (%) (experimental conditions: lipase amount 30 U; washing temperature 37°C; Washing time 30 min). All values are represented as mean ± sd of three replications.

Effect of different commercial detergents on oil removal has been shown in Table 2. Among all detergents (0.3%), higher oil removal (52%) has been observed with Wheel and it has been chosen for subsequent studies. In detail, the lipase was more effective with nonionics than with anionics which attributes to the higher enzyme activity inhibition by anionic surfactants [19, 20]. The relation between the Conc. of surfactant along with lipase and oil removal has been shown in Figure 3. At any detergent (Wheel) Conc. the oil removal with BDL solution has been found to be higher than with BD solution, which shows the advantage of lipase inclusion in the detergent formulation. Based on the present results, the usage of *S. arlettae* lipase with 0.5% Wheel improves the oil removal from soiled cotton fabric by 37% (B + D)–49% (B + D + L) utilizing 30 U of lipase at 37°C in 30 mins. of (Figure 3), and forecasting the lipase ability to use as an detergent additive. 

### 3.4. Effect of Lipase Amount on Oil Removal

**Figure 4:**
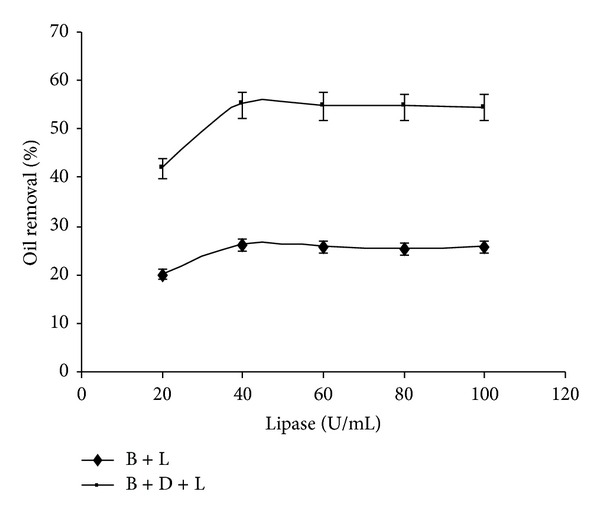
Effect of lipase amount on oil removal (experimental conditions: detergent concentration 0.5%; washing temperature 37°C; washing time 30 min). All values are represented as mean ± sd of three replications.

The relation between lipase concentration and oil removal has been depicted in Figure 4. In both cases, oil removal increases with lipase concentration till attaining the equilibrium state at a concentration of more than 40 units. The equilibrium attainment after certain lipase concentration depends on the initial rate of hydrolysis of triglyceride by lipase based on the interface area between insoluble triglyceride and aqueous solution of lipase. The surface area of a given amount of olive oil will be constant after a certain concentration of lipase with which interface is saturated. As shown in Figure 4, the addition of the lipase brought an improvement from 26% to 55% without the detergent and with detergent. Enhanced results of lipase in combination with commercial detergent have been also reported in case of *Pseudozyma* sp. NII 08165 [19] and *Pseudomonas aeruginosa* lipases [21].

### 3.5. Effect of Washing Temperature on Oil Removal

**Figure 5:**
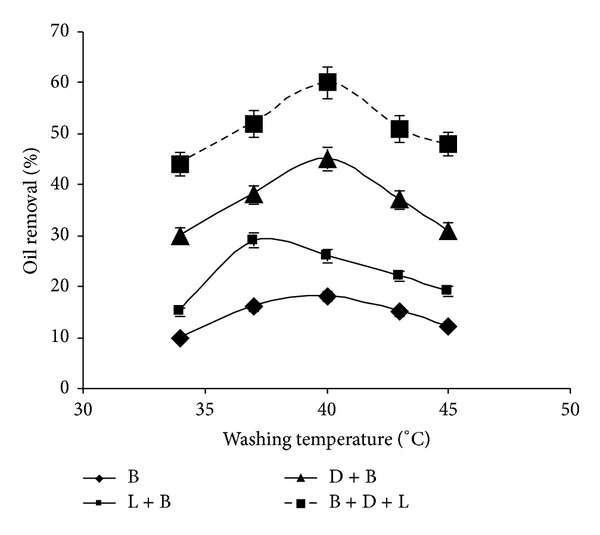
Effect of washing temperature on oil removal (experimental conditions: detergent concentration 0.5%; lipase amount 40 U; washing time 30 min). All values are represented as mean ± sd of three replications.

The results of oil removal with and without lipase at different temperatures have been shown in Figure 5. A maximum oil removal has been noticed in case of B-L washing solution at 37°C. In case of B-D solution, it has been observed that higher washing temperature is a prerequisite for better oil removal. [22, 23]. Higher oil removals have been observed with B-D-L washing solution compared with other washing solutions at any tempearture. The lipase contribution for higher oil removal has been observed at 40°C. 

### 3.6. Effect of Washing Time on Oil Removal

**Figure 6:**
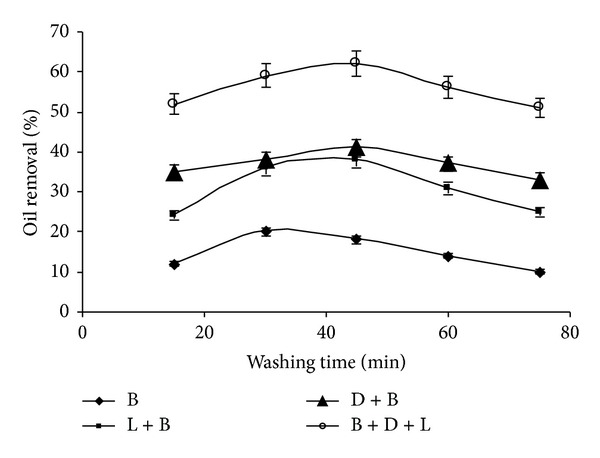
Effect of washing time on oil removal (experimental conditions: detergent concentration 0.5%; lipase amount 40 U; Washing temperature 40°C). All values are represented as mean ± sd of three replications.

The effect of washing time on oil removal has been depicted in Figure 6. It has been observed that on longer washing time only B-L solution worked properly for oil removal from soiled fabric. The oil removal using B-D solution seems to be constant 20 min afterwards. Finally, it has been observed that utilization of B-D-L solution results in enhanced oil removal (62%) using 45 min washing cycle. The significant contribution of lipase towards oil removal has also been observed with prolonged washing time. Fabric wash analysis of biosurfactant from *Pseudozyma* sp. NII 08165 revealed that the stain removal is more with the increased wash time [19]. Similar enhancement with increasing wash time has also been reported by Grbavčić et al. in case of *Pseudomonas aeruginosa* lipase biodetergent study [21].

## 4. Conclusions

Lipase from *S. arlettae* JPBW-1 was an ideal candidate for use in laundry detergent formulations, since it possessed better stability with surfactants, commercial detergents, and oxidizing agents. The additive effect has been observed more with nonionic surfactant than the anionic one's making it a novel lipase for further commercial utilization as a potential additive in detergent formulations. The results of this study show that lipase from *S. arlettae* improves the oil removal from soiled cotton fabric by 21% as an additive to commercial detergent, namely, 0.5% wheel under optimum conditions of 40 U of lipase in Tris-HCl buffer (0.1 M, pH 8.0) as washing temperature and washing time seems to be equal to 40°C and 45 mins, respectively. Hence, lipase from *S. arlettae* has been the ideal choice for formulating a environmentally friendly detergent formulation with the objective of triglyceride soil removal from fabrics. 

## Figures and Tables

**Table 1:** Washing solutions and its composition for making 100 mL of washing solution.

Components	Volume (mL)			
	B^a^	B^a^ + L^b^	B^a^ + D^c^	B^a^ + D^c^ + L^b^
Tris-HCL (0.1 M, pH 8.0)	40	40	40	40
Detergent (0.5%)	—	—	50	50
Lipase (50 U/mL)	—	10	—	10
Distilled water	60	50	10	—

^a^Buffer; ^b^lipase; ^c^detergent.

**Table 2:** Effect of lipase on removal of olive oil from cotton fabric with various detergents.

Detergents	Oil removal (%)	
	B^a^ + D^b^	B^a^ + D^b^ + L^c^
Tide	39.0	50.2
Rin Magic	33.2	45.8
Surf Excel	36.4	47.5
Ariel	31.2	43.0
SDS	30.6	39.5
Tween 80	28.3	38.0
Active Wheel	41.5	52.0
Nirma	30.2	41.2

^a^Buffer; ^b^detergent; ^c^lipase.
